# Synthesis and characterization of dextran-coated iron oxide nanoparticles

**DOI:** 10.1098/rsos.171525

**Published:** 2018-03-21

**Authors:** Andra Mihaela Predescu, Ecaterina Matei, Andrei Constantin Berbecaru, Cristian Pantilimon, Claudia Drăgan, Ruxandra Vidu, Cristian Predescu, Victor Kuncser

**Affiliations:** 1 University POLITEHNICA of Bucharest, 313 Splaiul Independentei, Bucharest 060042, Romania; 2 Department of Chemical Engineering and Materials Science, University of California Davis, One Shields Avenue, Davis, CA 95616, USA; 3 National Institute of Materials Physics, Atomistilor Street 405A, Magurele, Ilfov 077125, Romania

**Keywords:** magnetic nanoparticles, organic compounds, environmental applications, characterization techniques

## Abstract

Synthesis and characterization of iron oxide nanoparticles coated with a large molar weight dextran for environmental applications are reported. The first experiments involved the synthesis of iron oxide nanoparticles which were coated with dextran at different concentrations. The synthesis was performed by a co-precipitation technique, while the coating of iron oxide nanoparticles was carried out in solution. The obtained nanoparticles were characterized by using scanning electron microscopy (SEM), transmission electron microscopy (TEM), X-ray diffraction spectrometry, Fourier transform infrared spectroscopy and superconducting quantum interference device magnetometry. The results demonstrated a successful coating of iron oxide nanoparticles with large molar weight dextran, of which agglomeration tendency depended on the amount of dextran in the coating solution. SEM and TEM observations have shown that the iron oxide nanoparticles are of about 7 nm in size.

## 1. Introduction

Discharge and disposal of hazardous pollutants are governed by strict regulations. Nowadays, due to the increase in industrial manufacturing, the regulations for discharge of hazardous pollutants has undergone critical changes. A smart approach in the implementation of novel technologies needs to transform wastewater treatment technologies and to establish new criteria and design procedures to meet the ever-increasing demand. Therefore, innovative, safe and cost-effective strategies are required to reduce toxicity in the rapidly developing mining, paper, battery, pesticide and fertilizer industries. Nanotechnology has already proved to be an effective way to treat wastewaters [1,2]. Nanomaterials possess high adsorptive capabilities and may be used effectively to remove heavy metal ions from wastewaters. When magnetic nanoparticles are used, they can capture and transport heavy metal ions, being controlled by an applied magnetic field and acting as efficient vehicles to separate pollutants from wastewaters [3].

Generally, magnetic nanoparticles are synthesized by classical co-precipitation methods, and then coated with different organic compounds, thus conferring both magnetic and biocompatible properties to the final products. The nanoparticles can be used in environmental engineering, as adsorbent particles for metal ion removal [4].

Concern for the environment has become more and more significant in our lives, which increases the responsibility of researchers to search for applicable solutions in environmental protection. The progress of nanotechnology in the field of environmental engineering, especially in wastewater treatment, has opened new directions by applying advanced technologies to materials and devices. The removal of heavy metals from wastewaters using iron nanoparticles has been a good solution owing to their high efficiency and low cost. Nanoparticles can be introduced into wastewaters that are contaminated with heavy metals, and because of their magnetism and nano size they are able to quickly adsorb the heavy metals on their surface. The recovery of these loaded nanoparticles can be performed by applying a non-uniform magnetic field.

The literature indicates only few studies regarding the use of iron oxide nanoparticles coated with dextran for environmental applications. Among these, the main use is as a draw solute used for brackish water desalination using forward osmosis, as was described by Bai *et al*. [5,6], or as a material with magnetic and catalytic properties, especially as nanorods for bacterium detection [7].

Although iron oxide magnetic nanoparticle synthesis has advanced in the past years, their stability, agglomeration and loss of magnetization are still challenging problems. One of the solutions was to coat iron nanoparticles with a layer impermeable to oxygen, ensuring that oxygen does not reach the nanoparticle surface and change the magnetic properties [8]. Organic compounds, including surfactants and polymers, represent a good choice for coating the magnetic iron nanoparticles.

Among them, dextran has the advantage of being a biocompatible, biodegradable and water-soluble material [9–13]. Dextran is a complex branched polysaccharide polymer chain unit of various lengths, from 1000 to 2 000 000 Da. Dextran is a biocompatible material extensively used in biomedical applications for coating nanoparticles to prevent the agglomeration and toxicity of magnetic particles [14,15]. Dextran has been used to design dextran-coated nanomaterials with defined biological interactions with the complement system, which are able to either select complement activation pathways or prevent activation of the complement system on a rational basis [16]. *In vitro* effect of dextran-coated magnetic nanoparticles was tested on human colon cancer cell lines and the results showed that the dextran-coated nanoparticles had better biocompatibility compared with the uncoated nanoparticles [17].

Because of its biocompatibility properties, dextran was used in wastewater treatment applications to increase the activity of certain metallic oxides. Flower-like zinc oxide nanostructures were synthesized by a dextran-assisted solution technique [18]. This particular flower-like ZnO nanomaterial exhibits higher photocatalytic activity than ZnO fragments obtained without dextran.

Dextran was used in biodegradable blend membranes of poly(butylene succinate)/cellulose acetate/dextran as an additive to improve hydrophilicity, mechanical strength, biodegradability and antifouling properties of the membranes used in dairy wastewater treatment [19]. The report demonstrated that contact angle was reduced up to 16% with the increase in the dextran concentration from 0 to 2 wt%, while the amounts of pure water flux and permeate flux of wastewater were improved by 154% and 1543%, respectively.

Dextran has also contributed to the synthesis of novel biopolysaccharide derivatives as eco-friendly flocculants, which are needed for environmental protection [20]. Polysaccharide-modified flocculant is a remarkable material in the field of wastewater treatment, which can handle industrial effluent and sanitary sewage.

**Figure 1.**
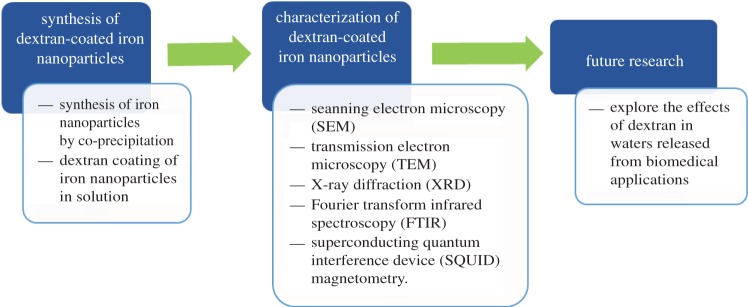
Illustration of the objectives of the research.

The aim of this study is to synthesize iron oxide nanoparticles (figure 1), coat them with 200 000 Da dextran and then characterize them for their possible application in the treatment of wastewaters coming from the biomedical field. These wastewaters can be treated using a magnetic module that was developed initially by our group to selectively remove heavy ions, biological material, cations, anions or other elements of interest from liquids via magnetic separation of nanoparticles [4].

The novelty of this research involves obtaining an eco-friendly material with promising properties for environmental applications, combining two types of materials: one used as flocculant (dextran) and the other with adsorbent and magnetic properties (iron oxide nanoparticles). Detailed synthesis and characterizations of the dextran-coated iron oxide nanoparticles are presented. Future work will be directed to assess the removal efficiency of certain bacteria and other various pollutants.

## 2. Material and methods

**Figure 2.**
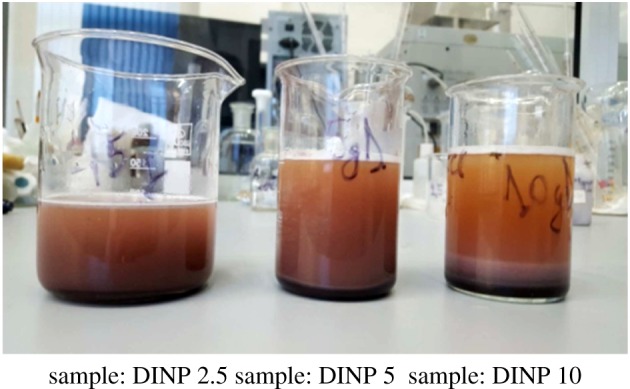
Illustration of the dextran-coated iron oxide nanoparticle solutions.

The synthesis of iron oxide nanoparticles was carried out using a controlled co-precipitation method, by mixing ferrous ion (Fe^2+^) and ferric ion (Fe^3+^) in alkaline solution as was described in our previous work [21], based on the Massart method [22]. The obtained suspension was centrifuged at 1200 r.p.m. for 5 min in order to obtain the final product as a dark-brown precipitate (figure 2). After centrifugation, separation was made using a magnet to hold the powder onto the vial wall and after this the precipitate was washed with distilled water until the wash solution reached a pH of 7. Then, the collected final product was dried in an oven at 60°C.

The dextran solution was prepared by mixing 2.5, 5 and 10 g of dextran with a molecular weight of 200 000 g mol^−1^ with 100 ml of deionized water. The respective solutions were put in contact with 1 g of iron nanoparticles at 100°C for 1 h in order to achieve coating of the iron nanoparticles with dextran. After the solutions were cooled at room temperature, in order to separate the newly synthesized materials, the suspensions were centrifuged at 800 r.p.m. for 15 min. The final products were separated using a magnet and then washed with methanol. The samples were labelled as follows: DINP 2.5 (1 g of iron nanoparticles coated with 2.5 g dextran), DINP 5 (1 g of iron nanoparticles coated with 5 g dextran) and DINP 10 (1 g of iron nanoparticles coated with 10 g dextran).

The structure, morphology and magnetism of the iron nanoparticles coated with dextran were investigated using X-ray diffraction (XRD), scanning electron microscopy (SEM), transmission electron microscopy (TEM), Fourier transform infrared spectroscopy (FTIR) and superconducting quantum interference device (SQUID) magnetometry.

The morphology and estimation of the sample sizes via SEM analysis was performed using a scanning electron microscope, QUANTA INSPECT F-type, with a field emission gun. For validation of the SEM results, TEM analysis was carried out using a TECNAI F30 G2 high-resolution electron microscope with 1 Å line resolution equipped with an X-ray dispersive energy (EDS) detector with 133 eV resolution. In order to establish the amorphous or crystalline nature of the samples, the XRD analysis was conducted using a PanalyticalX'Pert PRO MPD X-ray diffractometer with high-intensity Cu–K*α* radiation (*λ *= 1.54065 Å) and 2*θ* ranging from 10° to 90°.

Concerning the presence of the organic compound on the surface of the iron nanoparticles, a FTIR spectrometer Thermo Fischer Model Nicolet 50, with 0.09 cm^−1^ resolution and variable aperture, was used. Also, the samples were investigated with respect to their magnetic properties by SQUID magnetometry via the sensitive reciprocal space option (RSO).

## 3. Results and discussion

### 3.1. Scanning electron microscopy observation

**Figure 3.**
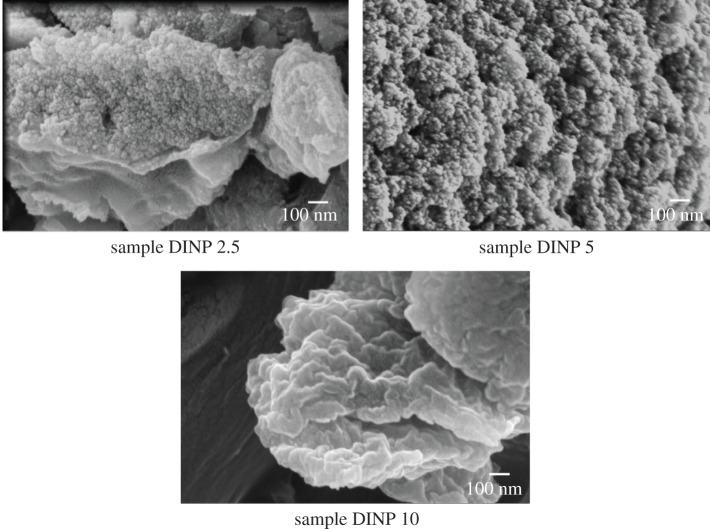
SEM images of the dextran-coated iron nanoparticles for various dextran concentrations.

Figure 3 presents the SEM observation of the iron oxide nanoparticles coated with 2.5, 5 and 10 mg dextran (labelled as DINP 2.5, DINP 5 and DINP 10, respectively). To prepare a sample for SEM analysis, a small amount of each sample was coated with 5 nm of Au. The SEM images show that the dextran-coated iron nanoparticles are agglomerated. However, it is not clear if dextran coated each nanoparticle or an agglomeration of nanoparticles, as in super-paramagnetic iron oxide nanoparticles (SIOPN). Agglomerated iron nanoparticles are often observed in the powders obtained by co-precipitation. However, the dextran layer should increase nanoparticle size, narrow particle size distribution and improve dispersion.

In order to understand the agglomeration in dextran-coated nanoparticles, we first checked if there was enough dextran in the coating solution, which might account for particle agglomeration. A simple calculation was performed to estimate the amount of dextran necessary to coat all of the 9.1 × 10^23^ nanoparticles of 7 nm in diameter in 1 g of iron oxide nanoparticles. Assuming a Stokes radius of 25 nm for a dextran molecular weight of 200 000, we need at least 8 × 10^25^ dextran molecules. Calculations show that in a solution of 2.5 g dextran, there are 2.5 × 10^27^. The number of dextran molecules in the solution that contains the least amount of dextran is actually much higher than that required to coat each nanoparticle, which indicates that the dextran amount in the coating solution is not a limiting factor in the DINP agglomeration.

We performed additional EDS measurements to better understand the distribution of the elements in the DINPs. The composition of the iron oxide nanoparticles coated with various amounts of dextran is presented in table 1. Comparing the three samples, we observed that with increasing dextran concentration of the coating solution, the amount of C increases while the amount of Fe decreases. These results indicate an increase in particle dispersion with the increase of dextran concentration in the solution, which may also indicate an improvement in dextran coverage on each nanoparticles.

**Table 1.** EDS analysis of the dextran-coated iron oxide nanoparticles.

element (wt%)	DINP 2.5	DINP 5	DINP 10
C–K	13.8	25.3	51.1
O–K	24.8	32.4	36.6
Na–K	4.2	3.3	0
Au–M	2.1	0.2	2
Fe–K	55.2	36.9	10.2

**Figure 4.**
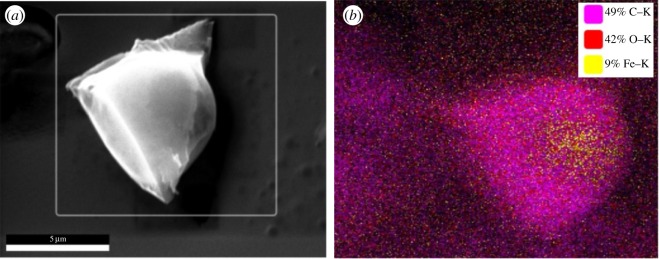
Element mapping showing a dextran-coated agglomeration of the dextran-coated iron nanoparticles ((*a*) SEM and (*b*) EDS).

However, it is still not clear if the iron nanoparticles are first agglomerated and then coated with dextran or if the dextran-coated iron oxide nanoparticles are agglomerated. As the stability and the magnetic properties of DINPs are affected by coating, we performed elemental mapping observation of an agglomeration of about 3–4 µm (figure 4). At this scale, the elemental distribution of C, O and Fe indicates that the dextran coats both nanoparticles and agglomerations of nanoparticles.

### 3.2. Transmission electron microscopy observation

**Figure 5.**
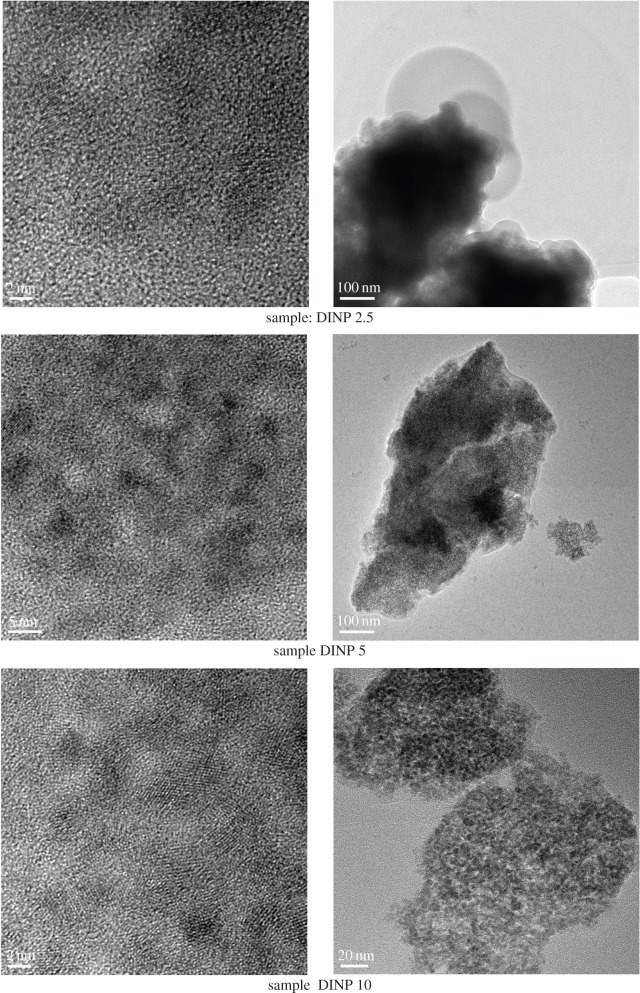
HRTEM (left) and TEM (right) images of the dextran-coated iron nanoparticles at various dextran concentrations.

TEM was used to observe the morphology, the quality of surface coating of nanoparticles and the dispersion of nanoparticles. High-resolution TEM (HRTEM) was also used. Figure 5 shows the TEM images of the iron oxide nanoparticles coated with 2.5, 5 and 10 g of dextran.

The morphology of dextran-coated nanoparticles is shown in figure 5. Magnetite particles were clearly seen as dark regions in the micrograph because their electron density is higher than that of the dextran component, giving a good contrast in the TEM image. The DINP 10 sample shows very clear crystalline structure. The magnetite particles have an average diameter of about 8 nm, which agrees with the SEM observation (figure 3).

TEM images of DINP 10 indicated a more uniform coating, which means a better dispersion of iron oxide nanoparticles, while in DINP 5 and DINP 2.5 the images presented an agglomeration tendency. There were a few coalesced nanoparticles that could be due to the fine coating of dextran. Also, the TEM image indicated that the dispersion of the dextran-coated iron oxide nanoparticles improves with an increase in dextran concentration in the coating solution.

According to the literature, natural polymers such as dextran influence extensively the stability of iron oxide nanoparticles because of their molecular weight and functional groups. Owing to their shielding properties, dextran controls the degradation process, the encapsulation efficiency and release rates [23,24], especially when the DINPs are used as an MRI contrast agent, for macrophage uptake and as a bioassay agent. TEM investigations indicated sizes between 6.5 and 50 nm for these applications [25–27].

Bai *et al*. [5] used DINPs for the first time in environmental studies as draw solute in order to ensure a good osmotic pressure for brackish water desalination. The DINPs were spherical, with a diameter of around 30 nm. In view of these observations, our results indicate that nanoparticles with an average size of about 7 nm could provide a better osmotic pressure if DINPs were used as draw solute for environmental applications.

### 3.3. X-ray diffraction

**Figure 6.**
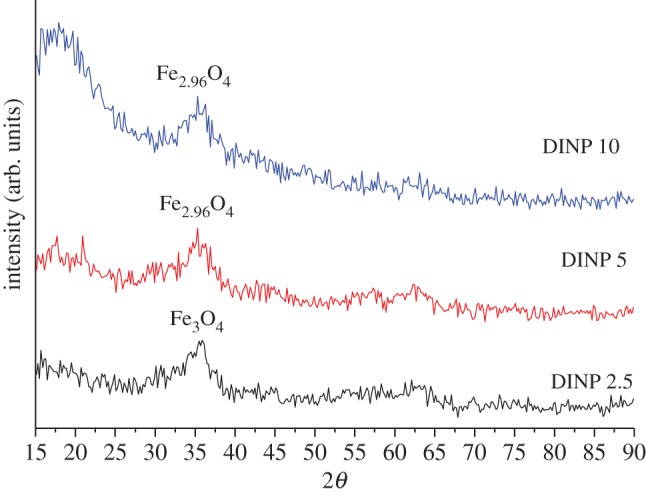
XRD imaging of the dextran-coated iron nanoparticles at various dextran concentrations.

Figure 6 presents the XRD patterns of the iron nanoparticles coated in solutions of various dextran concentrations. The analysis of the XRD patterns indicates the amorphous/crystalline nature of the dextran-coated iron oxide nanoparticles. The characteristic peak of Fe_3_O_4_ at 2*θ* (311) was observed for all the dextran-coated iron nanoparticles. The presence of magnetite as a unique phase was confirmed for the DINP 2.5 sample by the presence of the characteristic peak with maximum intensity at 35.79° (reference to ICCD 01-073-9877). In comparison, the main intensity peak at 35.38° was observed for the DINP 5 sample that indicates the presence of Fe_2.96_O_4_, which was induced by the bond with dextran. The influence of dextran as coated compound can be seen as a background characteristic between 15° and 25°, indicating a partial amorphous character of the DINPs.

The average particle sizes calculated using Debye Scherer's formula for dextran coated particles were 2.2, 1.4 and 1.2 nm for DINP 2.5, DINP 5 and DINP 10, respectively. Higher dispersion associated with the increase in dextran concentration in the solution as observed by TEM (figure 5) induced a decrease in nanoparticle size due to a decrease in agglomeration tendency.

The XRD results indicate smaller dimensions in comparison with the SEM results. This can be due to the fact that XRD indicates the crystallite size (crystalline domains) according to the Debye–Scherrer formula which highlights the limit between crystallographic planes of the particles, and SEM indicates the particle size as agglomeration of many crystallites. In this way, our results demonstrate a good correlation between the SEM and XRD investigations regarding the particle sizes.

### 3.4. Fourier transform infrared spectroscopy observation

**Figure 7.**
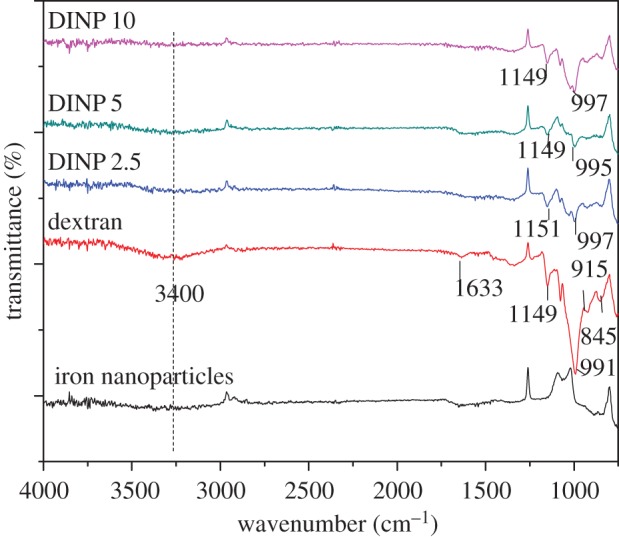
FTIR imaging of the dextran-coated iron nanoparticles at various dextran concentrations.

Figure 7 presents the FTIR spectra of the dextran-coated iron nanoparticles at various dextran concentrations, along with the dextran and the iron oxide nanoparticle spectra. The peak at 1149 cm^−1^ is caused by covalent vibrations of the glycosidic bridge. The peak at 997 cm^−1^ is due to the vibration of the C–O bond at the C-4 position of the glucose residue. The hydroxyl stretching vibration of the polysaccharide was caused by the band in the region of 2983–3612 cm^−1^. The band in the region of 1633 cm^−1^ was due to bound water. The band at about 1151 cm^−1^ is due to the stretching vibration of the alcoholic hydroxyl (C–O). These spectra also present the vibrational mode characteristics of the organic structure of dextran, such as the α-glucopyranose ring deformation modes at 845–915 cm^−1^ [28]; the presence of these vibrational modes is an important indication of surface functionalization [29].

These data demonstrate that the surface of magnetite nanoparticles has been covered with dextran polymer. It is believed that different interactions such as van der Waals force, hydrogen bonds and electrostatic interactions keep dextran on the surface of magnetite nanoparticles. These results were also validated by XRD investigations where an amorphous aspect detected between 15° and 25° indicated the organic nature of the coated samples.

### 3.5. Magnetism

**Figure 8.**
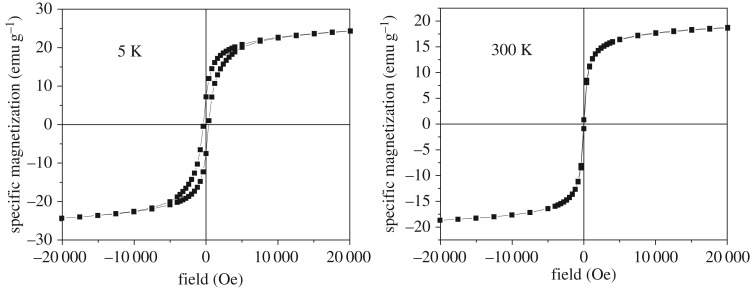
Hysteresis loops at 5 K and 300 K for sample Fe_3_O_4_ (naked nanoparticles).

**Figure 9.**
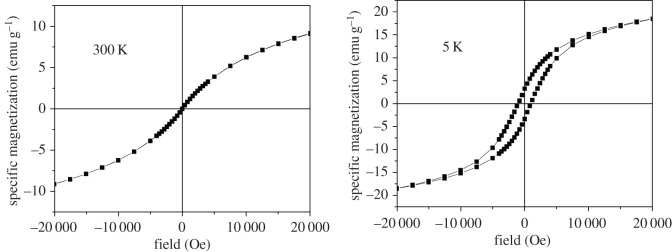
Hysteresis loops at 5 K and 300 K for sample DINP 2.5.

**Figure 10.**
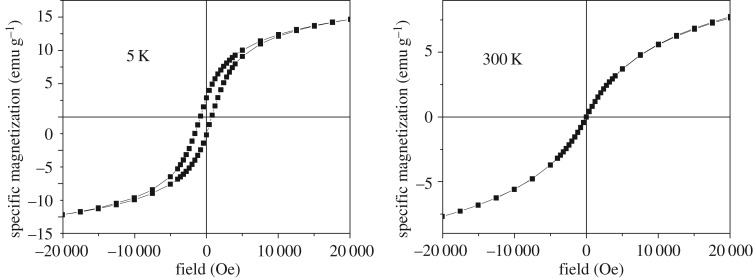
Hysteresis loops at 5 K and 300 K for sample DINP 5.

**Figure 11.**
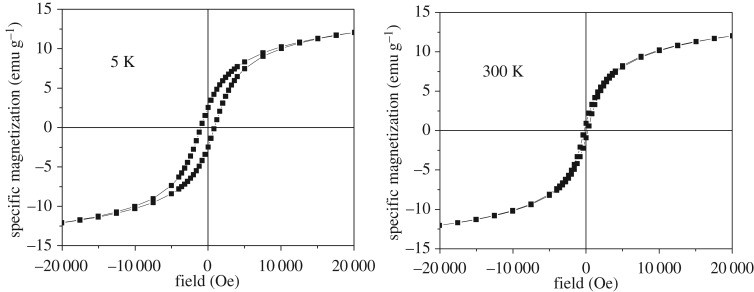
Hysteresis loops at 5 K and 300 K for sample DINP 10.

Naked magnetite nanoparticles (reference sample-code Fe_3_O_4_) prepared and dispersed in different amounts of dextran (e.g. with the sample-code DINP 2.5, DINP 5 and DINP 10, for increased amounts of dextran) have also been investigated with respect to their magnetic properties. Hysteresis loops collected at 5 K and 300 K for each sample are presented in figures 8–11.

A first aspect observed in figures 8–11 is the reduced (or even lack of) coercive field, as evidenced by the loops collected at 300 K in contrast to the ones collected at 5 K showing finite coercive fields of order of hundreds of Oe. This observation gives evidence for the presence of nanoparticles of reduced sizes (with super-paramagnetic behaviour at 300 K and blocking temperatures lower than 300 K). Taking into account an anisotropy constant specific to bulk magnetite and quasi-spherical shapes, nanoparticles with average sizes in the range from 8 to 18 nm can be concluded from such magnetic measurements.

The slopes of the magnetization curves in high fields (e.g. higher than 15 000 Oe) are almost constant and with almost similar values at both 5 K and 300 K. This is somehow unexpected in the assumption that a constant susceptibility would be specific to a paramagnetic/super-paramagnetic phase (e.g. at least at 300 K). In this case, the susceptibility will scale with temperature as 1/*T*, which means that a slope about 60 times higher at 5 K when compared with 300 K should be observed, which certainly is not the case. Therefore, the linear increase of the magnetization versus the applied field has to be due to a disordered magnetic structure of magnetite both at 5 K and at 300 K.

**Figure 12.**
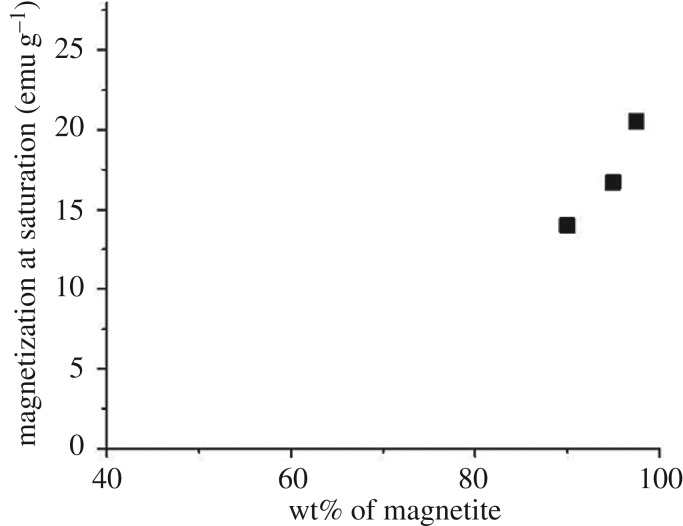
Variation of the saturation magnetization versus the percentage of magnetite in the sample.

As a consequence of the disordered ferromagnetic structure of magnetite, the saturation magnetization (approaching the spontaneous magnetization) of all samples has been calculated according to the well-known law of approach. In this way, values of 26.0(2), 20.5(2), 16.7(2) and 14.0(2) emu g^−1^ have been obtained for samples Fe_3_O_4_, DINP 2.5, DINP 5 and DINP 10, respectively. Of note, typical value of the spontaneous magnetization of bulk magnetite is about 90 emu g^−1^, but in this case even the reference sample Fe_3_O_4_ gives a much lower value (e.g. approx. 26 emu g^−1^) which can be explained only by a defect/disordered magnetic structure of the magnetite nanoparticles (which was already mentioned). This magnetic disordered state of each nanoparticle can be directly related to a disordered (amorphous like) crystalline structure, thus explaining the extremely low values of the structural coherence length (particle size) obtained by the Scherer formula. On the other hand, the saturation magnetization decreases strongly with respect to the increasing amount of dextran (e.g. in figure 12 is presented the variation of the saturation magnetization versus the percentage of magnetite in the sample), giving the possibility of estimating the relative weight of dextran to the magnetic material in each sample.

## 4. Conclusion

Dextran-coated iron oxide nanoparticles, named as DINPs, were synthesized in order to be used for future environmental applications. Iron oxide nanoparticles were obtained by co-precipitation method and then were coated with various dextran concentrations. The structure, size, morphology and magnetism of DINPs were studied and the main results are concluded as follows:

1. SEM investigations highlight a tendency of agglomeration for the iron oxide nanoparticles and an average size of about 7 nm. The size is validated by TEM observations which strengthens that the dextran coating of iron oxide nanoparticles affects the agglomeration tendency: the higher the dextran concentration, the better the dispersion of nanoparticles.
2. The XRD results shows that the crystallinity of iron oxide nanoparticles is only partially preserved during the coating process with dextran, but amorphous features become stronger as the dextran concentration increased.
3. FTIR results have shown that dextran coats the iron oxide nanoparticle surface and remains on the surface due to a combination of different interactions such as van der Waals force, hydrogen bonds and electrostatic interactions.
4. The composition of the dextran-coated iron oxide nanoparticles obtained by EDS has shown that the amount of Fe decreases as the C concentration increases in the coating solution.
5. An increase in dextran concentration in the solution results in an increase in particle dispersion that improves the dextran coverage on nanoparticles.
6. The magnetism results prove clearly that the dextran is strongly interacting with magnetite nanoparticles, changing their magnetic structure mainly at the nanoparticle surface where a strong magnetic disorder is induced.
7. The DINPs synthesized in this work will be tested as an eco-friendly material using its magnetic, catalytic and sensing properties for future environmental applications.

## Data accessibility

The relevant data for the experimental reproduction are thoroughly available in the Material and methods section. There is no additional raw or supporting data associated with the manuscript.
